# Preserved visuoconstruction in patients with Alzheimer's pathology and anti-neural autoantibodies: A case control study

**DOI:** 10.3389/frdem.2022.975851

**Published:** 2022-12-20

**Authors:** Niels Hansen, Sina Hirschel, Bianca Teegen, Jens Wiltfang, Berend Malchow

**Affiliations:** ^1^Department of Psychiatry and Psychotherapy, University Medical Center Göttingen, Göttingen, Germany; ^2^Translational Psychoneuroscience, University Medical Center Göttingen, Göttingen, Germany; ^3^Clinical Immunological Laboratory Prof. Stöcker, Lübeck, Germany; ^4^German Center for Neurodegenerative Diseases (DZNE), Göttingen, Germany; ^5^Neurosciences and Signaling Group, Institute of Biomedicine (iBiMED), Department of Medical Sciences, University of Aveiro, Aveiro, Portugal

**Keywords:** Alzheimer's disease, neural autoantibodies, autoimmunity, visuoconstruction, temporoparietal region

## Abstract

**Background:**

Alzheimer's disease (AD) is seldom reported to be associated with neural autoantibodies apart from those involved in axonal neurodegeneration and amyloidopathy in prior studies. Nevertheless, this is an under-investigated aspect of AD. As we do not know whether additional screening for autoantibodies in AD patients has additional diagnostic and therapeutic value, this study aims to shed light on whether visuoconstructive or figural memory capacities might distinguish these patient populations.

**Methods:**

In this pilot case series, we investigated eight patients suffering from cognitive impairment associated with cerebrospinal fluid (CSF)-based Alzheimer pathology (AP) and with verified anti-neural autoantibodies (AP Aab+) compared to eight AD patients presenting no autoantibodies (Aab–) (AD Aab–). Patients files were reviewed retrospectively regarding their neuropsychological profile assessed *via* the CERAD (Consortium to Establish a Registry for Alzheimer's Disease) test battery and psychopathology measured by the AMDP (Manual for the Assessment and Documentation of Psychopathology in Psychiatry) system. We also relied on diagnostic parameters as in the CSF and magnetic resonance images.

**Results:**

All patients shared the same pattern of dysfunctional word-list learning and word-list recall resembling a hippocampus-dependent memory dysfunction. Furthermore, both patient groups revealed a CSF profile concurring with Alzheimer's disease. However, visuoconstructive capacity, but not figure recall was preserved in AP Aab+ patients, but not in AD Ab-patients with the shared hippocampus-based memory dysfunction. We observed no relevant differences between the AP Aab+ and AD Aab– groups in CSF cell-counts or intrathecal IgG synthesis. The relative frequency of hippocampal and focal atrophy did not differ either between AP Aab+ and AD Aab– groups.

**Discussion:**

Our pilot findings are encouraging us to conduct large-scale studies to replicate our discovery of preserved visuoconstruction in AP Aab+ patients with hippocampus-based memory dysfunction. The role of anti-neural autoantibodies is still not fully understood. The detection of these autoantibodies might imply another disease pathology that could be either neuroprotective or be affecting other brain regions, i.e., less pronounced disease activity in the right temporo-parietal regions mainly involved in visuoconstruction.

## 1. Introduction

Alzheimer's disease (AD) is an increasing challenge for health systems and society worldwide. AD is currently diagnosed by relying on extensive diagnostic procedures including biomarker sampling and phenotypic classification. Novel criteria suggest employing both biomarker levels from the CSF or neuroimaging, and clinical phenotyping (Dubois et al., 2021). Autoantibodies against myelin have been proposed to lead to hippocampus-based memory dysfunction in AD, and are potential biomarker candidates for early AD (Papuć et al., 2015). Recent investigations have reported that anti-glial autoantibodies can occur in AD (Lim et al., 2019), and that potassium voltage-gated channel subfamily A member 2 (KCNA2) autoantibodies may coincide with a CSF-based AD pathology (AP) (Timäus et al., 2021). Furthermore, anti-ataxia cayman type protein (ATCAY) autoantibodies are reportedly elevated in AD compared to normal controls (Shim et al., 2022). The frequency and role of anti-neural autoantibodies are unknown in AD, as they have not been systematically investigated. Our pilot case series is dedicated to initiating a novel direction in AD research targeting the possible coexistence of anti-neural autoantibodies in patients with AP. We focused on two specific cognitive functions in our study, namely (1) visuoconstruction and (2) figural memory, as they can be impaired early in the course of AD (Whitwell et al., 2008; Ahmed et al., 2016) and in anti-neural autoantibody-associated neuropsychiatric disease (Hansen et al., 2020a; Mueller et al., 2022). Visuoconstruction is a neurocognitive function that relies on various cognitive subfunctions such as fine motor skills, the ability to understand visuospatial relationships, executive, and planning skills (reviewed by Benton and Tranel, 1993). Different types of mild cognitive impairment can affect various aspects of visuoconstructive abilities (Ahmed et al., 2016). Our aim is therefore to investigate whether patients presenting anti-neural autoantibodies and affected by biomarker-based AD pathology, as well as frequent hippocampus-based memory impairment are less impaired in their visuoconstructive and figural memory functions than patients with classic AD but without anti-neural autoantibodies.

## 2. Methods

### 2.1. Classification of patients

In our gender- and age-matched observatory and retrospective cases series study, we investigated eight patients with biomarker-based AP and proven serum or CSF autoantibodies (AP Aab+), and eight other patients with biomarker-based AD, no proven serum and/or CSF autoantibodies, and typical Alzheimer's (AD Aab–). Patients were recruited retrospectively by screening patient files in our Department of Psychiatry and Psychotherapy between 2016 and 2021. Their AD profiles were diagnosed by screening for biomarkers, namely when phosphorylated tau protein 181 (p-tau181) was elevated and the ratio of amyloid beta peptides 42 (Aß42) and amyloid beta peptide 40 (Aß40) (Aß42/Aß40) was below the normative level of our normative data. These criteria concur with international consensus for diagnosing AD (Jack et al., 2018). In those patients with neural autoantibodies, we did not use the term AD as such, but chose the somewhat more neutral “AP.” All patients shared the feature of a hippocampus-based memory dysfunction concurring with the classical AD phenotype (Dubois et al., 2021). Thus, both groups' inclusion criteria were AD-typical CSF pathology as described above, fulfilling an AD-typical neuropsychological profile. Note that neural autoantibodies were an inclusion criterion for the AP Aab+ group, but an exclusion criterion for the AD Aab– group. This study concurs with the current Declaration of Helsinki, and was approved by the Ethics Committee of the University Medical Center Göttingen.

### 2.2. Brain magnetic resonance tomography

To assess brain atrophy, 1.5-T MRIs were carried out and visually evaluated in the Department of Neuroradiology, University Medical Center Göttingen. In patients on whom we had neuroimaging data, we performed 1.5-T MRI at these sequences: transverse T2-weighted turbo spin-echo imaging, T1-MPRAGE 3D, diffusion-weighted imaging and transversal susceptibility-weighted imaging. No patients underwent a systematic tumor search after the detection of autoantibodies *via* whole body positron emission tomography-computed tomography. Focal cortical atrophy was inspected visually by relying primarily on visual assessments of prominent cerebral sulci, and asymmetric and relative losses of brain volume compared to earlier images and the contralateral side. We applied the pattern of temporal and temporoparietal cortical atrophy described in AD patients to assess cortical focal atrophy in patients, as in the MRI studies by Fox et al. (1996a,b, 2001). Hippocampal atrophy was visually assessed by a radiologist if either (1) the structures of the inner hippocampus were blurred or (2) hippocampus volume was reduced, or (3) the hippocampal fissure was dilated. For this purpose, T2-weighted images of the hippocampus were studied in particular, as these sequences are more suitable for measuring hippocampal atrophy (Fischbach-Boulanger et al., 2018). Patients' vascular pathology and lesions corresponded to cerebral microangiopathy assessed by Fazekas et al. (1987) in our study.

### 2.3. Assessment of cognitive functions

Patient neuropsychology was assessed applying the Consortium to Establish a Registry for Alzheimer's Disease (CERAD) Plus test battery (Morris et al., 1989) in all patients, including testing visual-constructive skills and figural memory compared to a standard control population with specific age limitations. We calculated our *Z*-scores relying on normative data, and used CERAD software (CERAD-Plus) to generate the *Z*-score results without further analysis by our staff. The CERAD-Plus test battery includes several tests, such as figure drawing to assess constructive practice, word-list learning and recall, and word recognition to test verbal memory, figure recall to test figural memory as well as semantic word fluency, and the Boston naming test to test language ability. In addition, semantic word fluency was examined to assess executive function. Visuomotor function was examined using the trial making test part A (TMTA) and trial making test part B (TMTB) to assess visuomotor speed and executive function. Cognitive impairment was classified according to the Diagnostic and Statistical Manual of Mental Disorders fifth edition (DSM-5) dividing into minor and major neurocognitive disorder (American Psychiatric Association, 2013). Furthermore, we retrospectively assessed patient files for psychopathological features *via* the Manual for the Assessment and Documentation of Psychopathology in Psychiatry (AMDP) classification (Broome et al., 2017). We relied on autoimmune indicators apportioned *via* strong and mild autoimmune indicators from our recent overview describing neural autoantibodies in different psychiatric syndromes (Hansen et al., 2020b).

### 2.4. Tau protein and ß-amyloid markers

To measure neurodegeneration markers, we referred to these normative values: a concentration is considered non-pathological level if (i) tau protein: <450 pg/ml, (ii) phosphorylated tau protein 181 (ptau181) <61 pg/ml, (iii) ß-amyloid 42 (Aß42) >450 pg/ml and (iv) ratio Aß42/ Aß40 × 10: >0.5. To measure p-tau 181 and total tau protein (*t*-tau), we employed the commercial enzyme-linked immunosorbent assay (ELISA) from Fujirebio [INNOTEST hTAU-Ag; INNOTEST PHOSPHO TAU (181P)]. In addition, to assess the CSF bioprobes for Aß42, we used the commercially available INNOTEST^®^ β-AMYLOID (1–42) ELISA kit (Fujirebio), whereas for Aß40 we measured the commercially available ELISA from IBL [AMYLOID BETA (1–40)]. All normative data for individual biomarkers (p-tau181, tau, Aß42, Aß40 and the Aß42/Aß40 ratio) were derived from the in-house normative laboratory values from the Neurochemistry Laboratory, Neurology Department, University Medical Center Göttingen. Blood samples were removed at the same time as the lumbar puncture. These biomaterial probes were handled according to the standard protocol in the neurochemistry laboratory of the Neurology Department, University Medical Center Göttingen. According to the Reiber scheme, a disrupted blood-brain barrier is indicated by an increase in IgG and albumin concentrations in the CSF in the same relationship.

### 2.5. Neural autoantibodies

All the neural autoantibody testing procedures were done in the Clinical Immunological Laboratory Prof. Stöcker. Standard-immunofluorescence tests were carried out to seek specific commercial autoantibodies against intracellular targets such as amphiphysin, CV2, ANNA-3, Tr/DNER, glutamic acid decarboxylase (GAD65), Ma1/Ma2, SOX1, Ri, Yo, HuD, and Zic4. In addition, we conducted immunofluorescence tests to assess autoantibodies against cell-membrane surface antigens like amino-3-hydroxy-5-methyl-4-isoxazolepropionic acid receptors 1/2 (AMPAR1/2), anti-N-methyl-D-aspartate receptor (NMDAR), gamma aminobutyric acid B receptor (GABABR), dipeptidyl-peptidase-like 6 protein (DPPX), leucin rich glioma inactivated protein 1 (LGI1), contactin-associated protein 2 (CASPR2), and aquaporin 4. Furthermore, to determine other specific autoantibodies like glycine receptors, recoverin, gamma aminobutyric acid A receptor (GABAAR), potassium voltage-gated channel subfamily A member 2 (KCNA2), glial fibrillary acid protein (GFAP), Homer3, Inositol 1,4,5-Trisphosphate Receptor Type 1 (ITPR1), mGluR5, Neurexin 3alpha, Neurochondrin, Rho-GTPase activating protein 26 and flotillin 1/2, we utilized home-made immunofluorescent, non-accredited tests (LDT) from the Clinical Immunological Laboratory Prof. Stöcker. Specific cell-based assays were done for all autoantibodies except anti-myelin and ANNA3. Out of 30 autoantibodies tested, we detected only 9.

### 2.6. Statistics

Statistics were analyzed using SigmaStat (Version 11.0, Systat Software Inc.). Graphs were drafted by SigmaPlot (Version 11.0, Systat Software Inc.). We analyzed z-scores of CERAD, age of patients, CSF cell counts as well as neurodegeneration parameters *via* correction for multiple testing between groups (AP Aab+ vs. AD Aab–). Furthermore, we tested for differences in relative frequencies of psychopathology and some CSF parameters (blood-brain barrier disturbance or intrathecal IgG synthesis) by Fisher's exact test (AP Aab+ vs. AD Aab–). If the data was normally distributed, the student's *t*-test was applied. The data's normal distribution was checked *via* a Shapiro->Wilk test. If the data were not normally distributed, the Mann Whitney *U* test was applied. We made a Bonferroni correction for multiple testing of the two conditions studied in our hypothesis, namely investigating visuoconstruction and figural memory. The significance level was set to *p* < 0.05.

## 3. Results

### 3.1. Patient characteristics

The age of our eight AD Aab– patients compared to AP Aab+ did not differ (AP Aab+: 79.8 ± 2.1 years vs. AD Aab–: 73.4 ± 4.2 years, Table 1). Furthermore, the age at onset and at diagnosis did not differ between groups. All patients had a pathological p-tau 181 and a reduced Aß42/40 ratio as a neuropathological hallmark of AD, in line with the latest AD diagnostic guidelines (Jack et al., 2018; Dubois et al., 2021). Anti-neural autoantibodies in serum were detected in 7 patients in our AP Aab+ group (Table 2). Furthermore, we detected serum and CSF antibodies in three AP Aab+ group patients (Table 2). Six autoantibodies were detected in the CSF of 4 AP Aab+ group patients (Table 2). All patients in both groups revealed similar deficits in hippocampus-dependent learning and memory, together with impaired list learning and list recall (Table 1). Psychopathological features are also depicted in Table 1. Not present were any consciousness disorder, worries, or compulsions, hallucinations, or ego disturbances. Ego disturbances signify experiences contributing to a disturbed self-perception entailing the phenomena of derealization, depersonalization, thought broadcasting, thought withdrawal, thought insertion or other feelings of an alien influence. We conducted an extensive search for potential clinical features of as indicators for autoimmune involvement in both groups (AD Aab– and AP Aab+). We refer to the clinical features mentioned in our recent review (Hansen et al., 2020b). We would like to add that we did not follow a selection strategy when seeking these clinical features, although that is potentially useful, since specific features like aphasia are among the clinical characteristics of certain dementia syndromes, i.e., primary progressive aphasia in frontotemporal lobar degeneration. In the AP Aab+ group, we detected aphasia, mutism or dysarthria in 2/7 patients (28%) in one AP Aab+ group patient (1/7, 14%), and focal neurological deficits, paresthesia, and a tumor as another feature in another Ap Aab+ group patient (1/7, 14%). In the AD Aab– group, one patient exhibited abnormal movements (1/7, 14%). In addition, the same patient in the entire AD Aab– group revealed a focal neurological deficit (1/7, 14%). The autoimmune indicators absent in both groups were: autonomic disturbances, central hypoventilation, decreased consciousness level, epileptic seizures, faciobrachial dystonic seizures, hyponatremia, infectious prodrome, new-onset severe headache, adverse response to antipsychotic and antidepressant drugs, optic hallucinations, presence of malignant neuroleptic syndrome, dynamic course, early resistance to psychopharmacologic drug therapy, and fluctuating psychopathology. CSF parameters such as pleocytosis, albumin content, intrathecal IgG synthesis, a blood-barrier disturbance and neurodegenerative markers are described in Table 1. Diagnostic data such as MRI imaging differentiated as generalized atrophy, focal atrophy, hippocampal atrophy, or cerebral microangiopathy are shown in Table 1. We observed no relevant differences in the relative frequency of hippocampal and focal atrophy between the AP Aab+ and AD Aab– groups. In addition, the patients were suffering from no current cancer or neurological disease early in the stage of their actual disease.

**Table 1:** Clinical and demographic characteristics of patients.

**Parameter**	**AP Aab+**	**AD Aab–**	**Statistics**
**Demographic parameter**
Sex, females/all	4/8 (50%)	6/8 (75%)	0.61#
Age year	79.8 ± 2.1	73.4 ± 4.2	0.38#
Age of onset year	77.8 ± 2.2	70 ± 4.1	0.19#
**Psychopathology**
Orientation dysfunction	5/8 (62.5%)	4/8 (50%)	1+
Attentional dysfunction	7/8 (88%)	8/8 (100%)	1+
Memory disturbances	8/8 (100%)	8/8 (100%)	1+
Formal thought disorder	1/8 (13%)	2/8 (25%)	1+
Affective disturbance	1/8 (13%)	3/8 (38%)	0.57+
Drive and psychomotor disturbance	0/8 (0%)	3/8 (38%)	0.2+
**CSF**
Cell count (< 5 μg/L)	0.6 ± 0.3	1.1 ± 0.4	0.35*
Albumin mg/L	273 ± 28	234 ± 22	0.31*
Tau protein (< 450 pg/ml)	738 ± 92	739 ± 132	0.79*
P Tau protein 181 (< 61 pg/ml)	134 ± 17	95 ± 13	0.09*
Aß42 (>450 pg/ml)	634 ± 11	526 ± 54	0.10*
Aß40	12880 ± 1086	14624 ± 776	0.23*
Ratio Aß42/40 × 10 (>0.5)	0.43 ± 0.02	0.41 ± 0.02	0.11*
Blood brain barrier disturbance	0/7 (0%)	0/7 (0%)	1+
Intrathecal IgG synthesis	1/7 (14%)	0/7 (0%)	1+
**MRI**
Generalized atrophy	1/7 (14%)	3/6 (50%)	0.27+
Focal atrophy	5/7 (71%)	3/6 (50%)	0.59+
Hippocampal atrophy	2/7 (28%)	0/6 (0%)	0.46+
Cerebral microangiopathy	4/7 (57%)	2/6 (33%)	0.59+
**Neuropsychological testing**
Semantic fluency	**–**0.6 ± 0.5	**–**1.7 ± 0.5	0.07#
Boston naming	**–**0.8 ± 0.4	**–**0.7 ± 0.3	0.81*
List learning	**–**2.4 ± 0.8	**–**2.6 ± 0.7	0.84*
List recall	**–**2.0 ± 0.2	**–**2.7 ± 0.4	0.20*
List recognition	**–**0.9 ± 0.7	**–**2.2 ± 0.4	0.12*
Phonematic fluency	0.6 ± 0.5	**–**0.7 ± 0.4	0.49*
TMT part A	**–**0.8 ± 0.3	**–**0.7 ± 0.4	0.81*
TMT part B	**–**1.2 ± 0.7	**–**0.9 ± 0.4	0.72*

AP Aab+, Alzheimer's disease pathology and anti-neural autoantibodies; AD Aab–, Alzheimer's disease without anti-neural autoantibodies; CSF, cerebrospinal fluid; MRI, magnetic resonance imaging; P Tau Protein 181, phosphorylated tau protein 181; TMTA/B, trail making test A/B; y, years. The values are depicted as mean ± standard deviation. For laboratory data normal ranges are shown in brackets.

* T-test was used for statistical comparison. #Mann Whitney U test was used for statistical comparison. + Fisher's exact test was used for statistical comparison.

**Table 2:** Profiles of autoantibody subclasses in patients with Alzheimer pathology.

**Patients AP Aab+**	**Gender (F/M)**	**Autoantibody serum**	**Autoantibody CSF**
#1	M	KCNA2	0
#2	M	Recoverin	0
#3	F	Neurochondrin, Titin	Neurochondrin, Titin
#4	F	Myelin	0
#5	M	CASPR2	0
#6	F	0	Zic4, Yo
#7	M	GAD65	GAD65
#8	F	Titin	Titin

AP Aab+, patients with biomarker-based Alzheimer pathology and detection of serum or CSF autoantibodies; CASPR2, contactin-associated protein 2; CSF, cerebrospinal fluid; F, female; GAD65, glutamic acid decarboxylase 65; KCNA2, potassium voltage-gated channel subfamily A member 2; M, male.

### 3.2. Cognitive dysfunction

**Figure 1:**
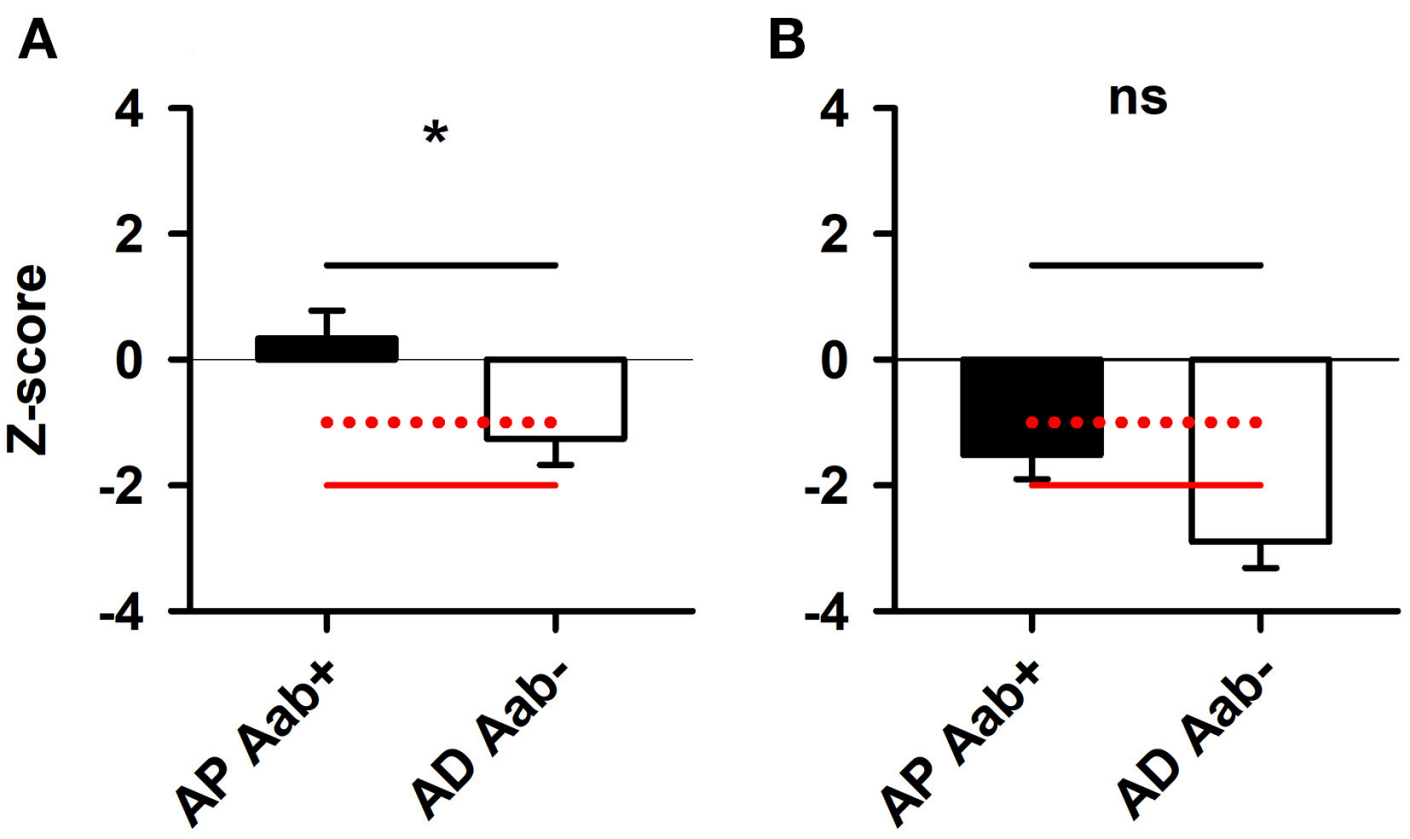
Preserved visuoconstruction but not figure recall in patients with Alzheimer's pathology and neural autoantibodies. **(A)** Visuoconstructive capacity was preserved in AP Aab+ patients (*n* = 8) **(A)** compared to AD Aab– patients (*n* = 8). **(B)** However, figure recall was not different between AP Aab+ patients (*n* = 6) compared to AD Aab– patients (*n* = 7). *p < 0.05 *t*-test. The red line at −2 *z*-score represents the double standard deviation from the norm values, while the dashed red line with the −1 *z*-score value indicates the single standard deviation from the norm values. A *z*-score value ≤-2 is considered severe cognitive dysfunction. In contrast, a *z*-score value of ≤-1 is considered mild cognitive dysfunction. AD Aab–, biomarker-based AD with no proof of serum and/or CSF autoantibodies; AP Aab+, with biomarker-based Alzheimer pathology and proof of serum or CSF autoantibodies; CERAD, consortium to establish a registry for Alzheimer's disease.

MMSE scores did not differ between groups, thus indicating a similar level of cognitive impairment in both groups (AP Aab+: 24 ± 0.9 MMSE score, AD Aab–: 23 ± 1.3 MMSE score, Table 1). Parts of the *z*-scores of the CERAD test battery subdomains are shown in Table 1. Visuoconstructive capacity was preserved in AP-Aab+ patients only (*p* < 0.05, Figure 1A)—they also demonstrated no loss of function in their visuoconstructural skills compared to the standard population in the CERAD test battery. However, figure recall did not differ between AP Aab+ patients from AD Ab– testing after multiple testing (Figure 1B). Additional cognitive functions such as semantic fluency, the Boston naming test, list learning, list recall, list recognition, phonematic fluency and the TMTA and B results did not differ either between AP-Aab+ and AD Aab– patients.

## 4. Discussion

Our study encourages research in a novel direction, namely to investigate the role of anti-neural autoantibodies in patients with AD pathology. The pathogenetic role of autoantibodies depends on their target, as intracellular antibodies often share a *T*-cell mediated mechanism, whereas in membrane-surface autoantibodies, they themselves play a major role in disease pathophysiology (Bien et al., 2012). 38% of the autoantibodies in patients in our case series are membrane-surface autoantibodies, which suggests that both these membrane-surface (such as CASPR2, KCNA2 and myelin antibodies in our cohort) and CSF intracellular autoantibodies (such as Yo, Zic 4, GAD65 antibodies in our cohort) might play a role in cognitive impairment when the CSF indicates AD pathology. We do not yet know whether these autoantibodies trigger neuropathological AD processes or even trigger AD mono- or co-pathology itself independent of brain-damaging processes related to autoimmunity. Although our sample is too small to draw conclusions, we observed a significant trend toward preserved visuoconstructive capacity in AP Aab+ patients. Visuoconstruction is a complex and multicomponential process involving different cortical brain regions. Impaired visuoconstructive capacity is detected in early AD (Martins-Rodrigues et al., 2021), in mild AD correlating with daily-living abilities (Fukui et al., 2009), and in genetic AD in conjunction with the M139V presenilin 1 mutation (Fuentes et al., 2021). The visuospatial abilities of AD patients might be impaired compared to healthy controls (Valencia and Lehrner, 2021). There is recent evidence that the visuoconstructive capacity (revealed as obtaining lower Rey-Osterrieth Complex-Figure-Test-c scores) was associated with hypometabolism in the temporo-parietal region (Beretta et al., 2021) suggesting that the AD pathology in our AD Aab– patients is probably more severe in their temporo-parietal region than in AD patients with AP Aab+. Another study showed that cognitive tasks related to visuoconstruction and visuospatial coordination correlate mainly with the right hemisphere (Hedderich et al., 2020) suggesting that AD Aab– patients are likely to exhibit relevant dysfunction in the right hemisphere's temporo-parietal region compared to AP Aab+ patients. The preservation of visuoconstruction in AP Aab+ patients might therefore depend on AD's neuropathological distribution pattern, thus affecting AD Aab– patients more strongly in the right temporo-parietal region. However, the cause of preserved visuoconstructive capacity remains an enigma, and this finding should first be replicated in a much larger cohort and then elucidated further in investigations enrolling large cohorts. The memory-consolidation capacity seems less affected in AP Aab+ patients (a non-significant trend we observed)—a finding that also deserves additional investigation in larger studies. However, these differences might also have to do with a more severe neuroinflammation that reveals other neuropsychological profiles, however, how this different pattern evolves is unclear. The stages of AD in our patient groups were not relevantly different in terms of the disease duration and onset-age of symptoms. Thus, their differences in visuoconstructive capacity cannot be attributed to another disease stage, as patients in both groups were mildly affected, and their onset-age did not differ.

### 4.1. Production of neuronal autoantibodies associated with Alzheimer‘s pathology

Neural autoantibodies are associated with various neurological diseases (Prüss, 2021). The molecular mechanisms of neural autoantibodies in neurological disease are diverse, and depend on autoantibody subclass and antigen location (Duong and Prüss, 2022). Our patients did not prove to be affected by any neurological disease other than AD pathology, i.e., traumatic brain injury or the presence of neurological disease at an early stage of their disease, as far as we know from their medical records. In addition, there were no common infectious conditions such as COVID19 or known current cancer. Considering that severe viral infection or cancer can often trigger the production of neural autoantibodies (Prüss, 2021), we think it possible either of these conditions could have been recently present, and we cannot rule them entirely out at the time of autoantibody testing. The neurodegenerative process involving both axonal damage and amyloid-ß pathology as supported by our CSF markers is rarely associated with processes of pure autoimmune disease like autoimmune encephalitis (Day et al., 2021). The question is whether the neurodegenerative process triggers additional autoantibody production, or whether an initial neuroglial inflammatory state involving autoantibody production might trigger long-term rather than transient neurodegeneration. Neural autoantibodies are not only detected in conjunction with neurological diseases. Several reports suggest that psychiatric syndromes may also have an autoimmune basis (Hansen et al., 2020a, 2022a; Endres et al., 2022). Therefore, it is extremely difficult to distinguish these primary psychiatric syndromes from secondary psychiatric syndromes. Psychopathology is one tool that could help (Grenzer et al., 2022); another option in dementia would be to assess visuoconstruction, which could help to distinguish AD patients in their dementia stage from those associated with neuronal autoantibodies.

### 4.2. Female predominance of specific CSF neural autoantibodies in patients with Alzheimer's pathology

It is possible that an autoinflammatory component is related to the neurodegenerative process. Previous work has shown that certain autoantibodies are associated with different AD stages like MCI and AD, such as anti-ATCAY-IgG or anti-PAIP2-IgG, known to be associated with a higher risk of MCI and AD (Shim et al., 2022). Moreover, in their study they detected the presence of anti-ATCAY IgM autoantibodies at each clinical AD stage, such as MCI and AD (Shim et al., 2022). Their results suggest that chronic exposure to the antigen is present in AD, regardless of clinical stage. The occurrence of such neuronal autoantibodies in AD could trigger neuroinflammatory processes or even initiate such a neurodegenerative process. Another aspect in this regard is the predominant identification of CSF autoantibodies in our female patients (Table 2), and published reports (Hansen et al., 2022b) were confirmed by a study by Shim et al. (2022), who also demonstrated serum autoantibodies in AD patients only in females and a female preponderance in patients with AD and antibodies to angiotensin-2 type 1 receptor (Giil et al., 2015). We do not know the underlying mechanism for a possible sex-dependent phenomenon of neuronal autoantibodies in AD, which could be related to exposure to specific antigens in females and depend on the autoantibody subclass. This suspicion should be confirmed in a study with a larger sample and more subjects of both sexes. In addition, how the Braak or Thal stage of AD is related to the presence of autoantibodies is of great importance.

### 4.3. Limitations

Our pilot case series' main limitation is its small sample size, which precludes any conclusions for clinical practice. The small number of subjects is an obvious study limitation. However, we had initially observed (and found fascinating) the preservation of visuoconstructive abilities in AP Aab+ patients. However, our findings encourage us to conduct a large-scale cohort study to investigate whether detecting a preserved visuoconstructive capacity can be replicated in other (and more) AP Aab+ patients. If so, this parameter might eventually prove to be a relevant differential-diagnosis instrument when investigating additional anti-neural autoantibodies. In addition, we think it would be fascinating examine in another study with a larger sample whether AD Aab– patients' deficient visuoconstructive ability is a gender-dependent or -independent phenomenon. An additional limitation is that our AP Aab+ group is heterogeneously restricted to the presence of diverse anti-neural autoantibodies. Autoantibody heterogeneity might also play a role in preserving or interrupting visuoconstructive abilities, an issue worthy of further investigation in a larger cohort with homogeneous autoantibody subclass groups. In addition, it would be worthwhile to shed light on another aspect, namely the association between ApoE4 carriers and autoantibody production in a prospective study with higher patient numbers, since a study by Shim et al. (2022) showed that more autoantibodies were detected in ApoE4 carriers than in non-ApoE4 carriers. The prevalence of neural autoantibodies in AD pathology is unclear, and prospective large samples will have to be recruited to answer that question. In addition, note the limiting factor that the heterogeneity of the existing autoantibodies can reveal a clinically highly diverse scenario, making it difficult to apply our findings to corresponding patients in a real-world scenario. Due to our study's retrospective character, missing data restrict the value of our MRI data. Further studies should also address the question of whether focal atrophy correlates with the presence of autoantibodies in AP Aab+ patients *via* a voxel-wise morphometric MRI examination. Another interesting aspect would be whether patients with autoantibodies and those without autoantibodies are in a limbic or isocortical (1) Braak neurofibrillary tangle (NFT) or (2) Thal stage. However, we were unable to conduct any postmortem examinations, and no (1) tau PET was available to establish the NFT Braak stage or (2) amyloid PET for Thal stage. MRI was not applicable for predicting Braak NFT stage in our retrospective study because it requires automatic brain segmentation and classification-based machine learning technique, which could not be accounted for in our retrospective *in vivo* MRI data that were not consistently available from all patients. Furthermore, MRI's predictive accuracy in detecting Braak NFT is only moderate (Dallaire-Théroux et al., 2019).

## 5. Conclusion

Our study data indicate functional neuropsychological differences in the visuoconstruction capacity of AP Aab+ vs. AD Aab– patients. These preliminary data should be replicated and investigated *via* neuropsychological and neuroimaging techniques in a larger and more homogeneous cohort presenting similar clinical phenotypes of hippocampus-based memory dysfunction but still revealing additional neural autoantibodies. The role played by neural autoantibodies in patients with AD pathology could thus be elucidated.

## Data availability statement

The raw data supporting the conclusions of this article will be made available by the corresponding author, without undue reservation.

## Ethics statement

The study involving human participants was reviewed and approved by Ethical Committee of the University Medical Center Göttingen. Written informed consent for participation was not required for this study in accordance with the national legislation and the institutional requirements. Written informed consent was obtained from the individual(s) for the publication of any potentially identifiable images or data included in this article.

## Author contributions

NH wrote the manuscript. SH, BT, JW, and BM revised the manuscript for important intellectual content. All authors contributed to the article and approved the submitted version.
